# Duty-Free Rectified Spirit in Hospitals

**Published:** 1914-11-21

**Authors:** 


### DUTY-FREE RECTIFIED SPIRIT IN HOSPITALS.

### Objections Answered and New Points Urged.

Two questions are involved in the case presented in The Hospital of October 31 last, p. 101. First, and less important to hospitals, the necessity of duty-free spirit, if the English manufacturer is to be put on an equal footing with Continental competitors; and secondly, the great help it would be to voluntary hospitals to be allowed duty-free spirit in the manufacture of tinctures, iodine, &c. Our attention has been called to the distinction drawn by the Inland Revenue authorities between (*a*) duty-free spirit in the manufacturing of galenicals, in which process the alcohol is not destroyed; and (*b*) in the manufacture of chloroform, ether, etc., in which processes the spirit is no longer present as such.

### Duty-free Spirit with Non-destruction of the Alcohol.

We can quite understand that a concession to galenical manufacturers of duty-free spirit in (*a*) would open a very wide door for the introduction of abuses, and in the diverse conditions in which in those circumstances the spirit would be used, observation by the Customs and Excise Departments, whose duties have recently been considerably added to, would be a very difficult matter. We are afraid this represents an obstacle to such a concession unless the manufacturers themselves can arrive at some basis which would enable them to satisfy the Commissioners' requirements, whatever these may be found to be. There is added encouragement for them to make application, as the Government have promised to consider the possibilities of allowing facilities for the introduction of new industries. With regard to a concession to hospitals, there is not, we think, the same objection, as there should be no difficulty in satisfying any conditions the Customs and Excise authorities may impose, and inasmuch as in hospitals the galenicals, etc., which we ask should be allowed to be made up with duty-free spirit are solely consumed by hospital patients, or used for hospital purposes on the prescriptions of medical men, the question of trading is not raised.

### Duty-free Spirit with Destruction of the Alcohol.

With regard to point (*b*), here, at least, the manufacturer and the hospital administrator are at one, and if we can only prove that part of our case, it is undoubtedly the most important part, and if those in a position to do so would suggest the best form in which the case of the hospitals and the manufacturers, either singly or jointly, could be submitted to the Inland Revenue authorities, we would gladly do our best to submit the considered views to the hospitals and the manufacturers, with a view to an application to the authorities, or in such other manner as may be advised. We say this in response to correspondents who hold that the service we might thus render would undoubtedly be to the great advantage of the hospitals.

### The Case for the Voluntary Hospitals.

But apart from the case of the manufacturer, that for the hospitals may be stated rather more fully than was possible in our article already mentioned. In the first place, a joint, rather than a series of individual applications should be made to the Inland Revenue authorities by the British hospitals, for the case for the hospitals would be strengthened by a united application on prearranged and well-considered lines. In support of the need for action we submit the case of a hospital (and there are doubtless many such) using, say, 100 gallons of rectified spirit per annum in the preparation of iodine, tinctures, etc. The cost of spirit at, say, 28s. per gallon would be £140, and of this approximately £120 represents duty paid to the Government. Our readers will readily recall the misgivings of hospital administrators when the Budget of 1909 was introduced, imposing an increased duty of 3s. 9d. per proof gallon on spirit, misgivings the effect of which was increased by an immediate advance of 6s. per gallon in the cost of rectified spirit, resulting at those hospitals using 100 gallons per year in an increased expenditure of £30, a charge which has been borne ever since. But in addition to this, and supporting the submission that the English manufacturer is disadvantaged, commercial commodities—*i.e.*, ether, chloroform, etc.—in which alcohol as such was no longer present were also immediately increased in price. Where before 1909 the price of ethylic chloroform was 5s. 8d. per lb., it at once advanced to 7s. per lb., while ether advanced from 7s. to 8s. 6d. per lb. In institutions of 250 beds and upwards the additional cost on these items would certainly not be less than £100 per annum. Not only, therefore, was the English manufacturer apparently placed at a positive disadvantage with his foreign competitors, but still further burdens were thereby imposed on British hospitals, amounting, there can be no doubt, to many hundreds of pounds per annum.

### English and Continental Hospitals.

While, however, the contention appears to be right that the manufacturers of tinctures, whether English or foreign, are on an equal footing, both being equally taxed, there is a fundamental disadvantage under which English hospitals work in comparison with Continental hospitals, and that is that a prohibitive duty always acts as a discouragement to research and inquiry. It may thus be submitted—we say " may " because we cannot at present find definite evidence that no duty is charged, although we believe such is the case—that the reason of chemical preparations being so extraordinarily advanced in Germany is to be found in the fact that experimental calculations and procedure are facilitated and encouraged by duty-free spirit, whereas in England they are handicapped by a duty six times as large as the original value.

Hence the cost of the same chemical preparations may be quite out of proportion in two different countries.

### Important National Services by British Hospitals.

For these reasons we submit that the British hospitals are seriously handicapped, and we suggest that this fact alone should be adequate reason for a combined application to the Inland Revenue authorities for concessions in their favour. Further, there are the more sentimental, but no less important aspects of the case, which should, we think, also be submitted. There has never been a time more favourable for an appeal, because there never has been a period when the British hospitals have rendered more useful and important national service than in the present crisis.

1. Throughout the United Kingdom there has been an unanimous readiness on the part of the hospital committees to place the greatest possible facilities at the disposal of the Government for the treatment of wounded soldiers, at a cost which must inevitably add to the burdens of institutional finances. The rate for subsistence suggested by the Government (2s. per day) is ridiculously out of proportion to the actual cost. The accommodation afforded is thus obviously at the cost of the civil population, who in providing the funds for the upkeep of the institutions must also bear the balance of the cost of treatment of wounded soldiers over and above the Government grants received.

2. In some institutions wards have been set aside for the treatment of would-be recruits rejected from the Services on account of physical disability—a great national gain which there is reason to suggest should not altogether be a charge upon the patriotism and good will by which such hospitals are actuated.

3. The military hospitals (Territorial) have been largely equipped from the staffs of the voluntary hospitals, and these hospitals have also had their ranks depleted by the withdrawal from the same service of fully trained nurses, while other fully trained nurses have been supplied to the Queen Alexandra's Imperial Military Nursing Service. Resident medical officers, and in some cases administrative officers too, have been given facilities to relinquish their appointments for service with the Imperial Forces. The voluntary hospitals have thus been deprived of their well-trained officers, and are, it cannot be gainsaid, endeavouring to perform their work effectively with less trained and experienced officers.

4. There can be no possible doubt, too, that this most effective service is being rendered at a time when funds are likely to be diminished by reason of the numerous war funds and the certain indication of increased taxation, which must inevitably have the result of withdrawing for a time moneys which might otherwise be available for hospital purposes.

### The Hospitals and the Inland Revenue Board.

In fact, if ever there were an occasion when the hospitals merited every possible encouragement and facility, it is the present. We therefore suggest, with conviction, that application be made to the Inland Revenue authorities under Section 8 of the Finance Act of 1902 (2 Edward VII., Chapter 7), or under such other Act as may be thought advisable, for hospitals to be included in the list of public institutions, universities, and colleges which are privileged to use duty-free spirit for the purposes of research and teaching.

